# Chemical Synthesis, Backbone Cyclization and Oxidative Folding of Cystine-knot Peptides — Promising Scaffolds for Applications in Drug Design

**DOI:** 10.3390/molecules171112533

**Published:** 2012-10-24

**Authors:** Michael Reinwarth, Daichi Nasu, Harald Kolmar, Olga Avrutina

**Affiliations:** Institute for Organic Chemistry and Biochemistry, Technische Universität Darmstadt, Petersenstraße 22, D-64287 Darmstadt, Germany

**Keywords:** CCK, cyclotide, cystine knot, ICK, inhibitor, knottin, miniprotein, native chemical ligation, oxidative folding

## Abstract

Cystine-knot peptides display exceptional structural, thermal, and biological stability. Their eponymous motif consists of six cysteine residues that form three disulfide bonds, resulting in a notably rigid structural core. Since they highly tolerate either rational or combinatorial changes in their primary structure, cystine knots are considered to be promising frameworks for the development of peptide-based pharmaceuticals. Despite their relatively small size (two to three dozens amino acid residues), the chemical synthesis route is challenging since it involves critical steps such as head-to-tail cyclization and oxidative folding towards the respective bioactive isomer. Herein we describe the topology of cystine-knot peptides, their synthetic availability and briefly discuss potential applications of engineered variants in diagnostics and therapy.

## 1. Introduction

Cystine-knot peptides, also termed knottins, are promising scaffolds for the design of peptide-based pharmaceuticals as they combine potent bioactivities with remarkable thermal and proteolytic stabilities [1,2,3]. Their amide backbone of approximately 30 amino acid residues is compacted by three disulfide bonds forming a characteristic ‘pseudo-knotted’ structure [4]. Cystine-knot peptides can be divided into three major subclasses: inhibitor cystine knots (ICK), cyclic cystine knots (CCK) and growth factor cystine knots (GFCK). While the majority of cystine-knot protease inhibitors comprises a linear backbone and displays inhibition constants in the low nanomolar to picomolar range, CCK peptides are defined by a head-to-tail backbone cyclization motif [5]. Compared to ICK and CCK, GFCK peptides are larger, less stable and commonly produced recombinantly. Hence, they will not be considered here.

The systematic research in the field of cystine-knot peptides arose in the early 1970s when a cyclotide kalata B1 was identified in Congo, where women brew tea from the leaves of the plant *Oldenlandia affinis* to accelerate childbirth [6]. Nevertheless, it took until the 1990s to finally solve the structural properties of CCK and a short period later also of ICK peptides [7,8].

To date, cystine-knot peptides have been found in diverse organisms: arthropoda, fungi, mollusca, plantae, porifera, and vertebrata [9,10]. Due to their wide-spread occurrence in combination with a structurally conserved core and an astonishing diversity with respect to amino acid sequence and function, they can be considered as one of Nature’s combinatorial libraries [11].

## 2. Structure

### 2.1. The Cystine-Knot Motif

**Figure 1:**
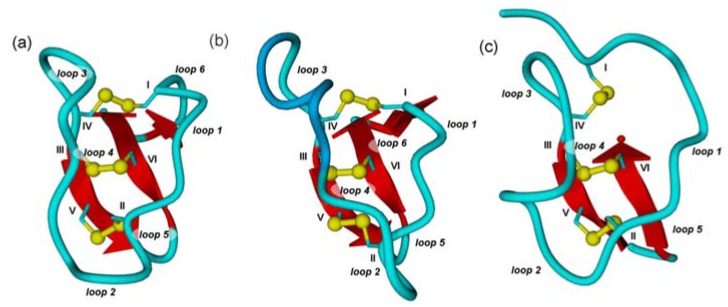
Cartoon diagrams of prototypical cystine knots. Loops are depicted in light blue and numbered according to their appearance in the sequence, α-helices in dark blue, β-sheets in red, and cysteines in yellow with Roman numerals according to their appearance in the sequence. (**a**) Möbius cyclotide kalata B1. (PDB-ID: 1NB1) (**b**) Bracelet cyclotide cycloviolacin O2. (PDB-ID: 2KNM) (**c**) Acyclic inhibitor cystine knot ocMCoTI-II (PDB-ID: 2IT8). Structures modeled with Yasara Ver. 12.4.1.

Cystine knots share common structural motifs that are defined by three antiparallel *β*-strands which are connected through short loops along with a considerable network of hydrogen bonds, and the eponymous knotted disulfide connections [12]. The constrained conformation mainly results from the disulfide bond between CysIII and CysVI (cysteines within the sequence are numbered according to their appearance from the amino- to the carboxy-terminus) which is threaded through the embedded ring formed upon the disulfide linkage of CysI and CysIV as well as CysII and CysV (Figure 1) [13].

Despite sequential and numerical differences within the variable loops of the different families, they all share the knotted core merging them into the cystine-knot family. These cystine motifs, in fact, are more important for peptide stability and rigidity than an eventual backbone cyclization [14]. Additionally, an extensive network of hydrogen bonds, especially *via* the β-sheets contributes an essential energetic value to the thermodynamic stability of cystine knots [11,12].

**Figure 2:**
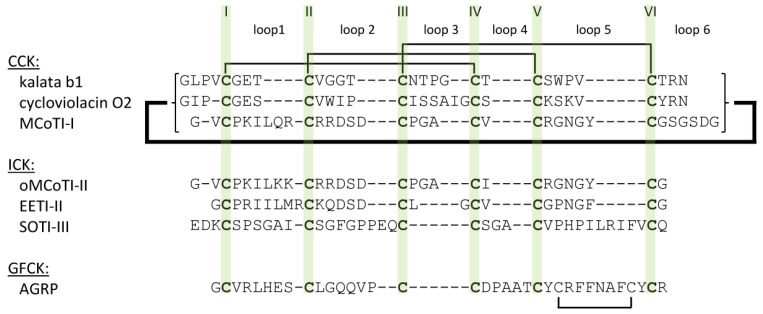
Sequence alignment of certain cystine knots. Cystine connections as well as head-to-tail macrocyclization motif are indicated.

These structural constraints leave the loops (Figure 2) in a surface-exposed state regardless of the hydrophobicity of the assembled amino acid residues. Therefore, also highly hydrophobic residues can be presented on the outer shell of the miniprotein targeting hydrophobic binding pockets or disrupting cell membranes [5,12].

### 2.2. Cyclic Cystine Knots

Cyclic cystine-knot peptides combine a macrolactam backbone with the knotted disulfide pattern [11]. They are supposed to play an important role in plant defense, as most of them have insecticidal activity due to their ability to disrupt cell membranes [15]. Interestingly, recent studies report antimicrobial, anti-HIV, and cytotoxic activities as well [16,17,18,19,20].

Structurally, cyclotides are divided into Möbius, bracelet, and trypsin inhibitor subclasses. In comparison to other cyclotide families cyclic trypsin inhibitors MCoTI-I and –II extracted from the seeds of *Momordica cochinchinensis* display considerable structural differences in their loop regions, obviously demonstrating similarities with knottins from the squash inhibitor family (Figure 2) [21,22,23]. Therefore, we share the opinion to categorize them into the ICK family [11,21].

The cyclotide kalata B1 is the prototypic cyclotide of a Möbius type, while cycloviolacin O2 is a common example for a bracelet cyclotide (Figure 1 and Figure 2) [11]. Conformational differences between Möbius and bracelet cyclotides are caused by the presence or absence of a *cis*-proline in loop 5. This moiety induces a twist in the orientation of the central *β*-sheet of Möbius CCK peptides, thus causing their oblate shape compared to bracelet cyclotides which lack this conformationally determinative unit [5].

### 2.3. Inhibitor Cystine Knots

ICK peptides, also referred to as knottins, are found in the seeds of various plants, among them bitter gourd *Momordica cochinchinensis* (MCoTI I-III), squirting cucumber *Ecballium elaterium* (EETI I-III), and spinach *Spinacia oleracea* (SOTI I-III) (Figure 1 and Figure 2). Their potent inhibitory effect against one of the major digestive proteases, trypsin, indicates their role in zoochory. While MCoTI and EETI are members of the squash inhibitor family with the inhibitory loop located between CysI and CysII, SOTI miniproteins display similarity to a class of antimicrobial peptides from the seeds of *M. jalapa* with CysV and CysVI flanking the inhibitory loop [8,21,24,25,26].

ICK peptides do not necessarily possess a cyclic backbone (indeed, only MCoTI-I and MCoTI-II are macrocyclic) but are defined according to their inhibitory effect against their respective target proteases. Cyclic trypsin inhibitors have been reported to be more potent than their open-chain counterparts. Nevertheless, inhibition constants of open-chain variants are still in a low nanomolar range [23]. Surprisingly, backbone cyclization only has minor effects on thermal and proteolytic stability providing evidence that the cystine knot motif is mainly responsible for the remarkable robustness of this scaffold [27].

## 3. Synthesis of Cystine-Knot Peptides

In this section we will critically discuss recombinant and chemical synthesis of cystine-knot peptides. Although the permanently increasing arsenal of reagents, methodologies, and instruments for solid phase peptide synthesis (SPPS) has largely eliminated synthetic problems during chain assembly, backbone cyclization and oxidative folding towards the regioselective formation of multiple disulfide bonds are still the crucial steps during total chemical synthesis of cystine knots and will therefore be considered in further detail [28,29,30,31,32,33].

### 3.1. Recombinant Production

In Nature, biosynthesis of cystine-knot peptides has been evolutionary optimized towards high yields of the bioactive forms [34]. Therefore, extraction of cystine-knot peptides from the corresponding plant sources is a common way to isolate the wild type sequences [31,35,36,37].

**Figure 3:**
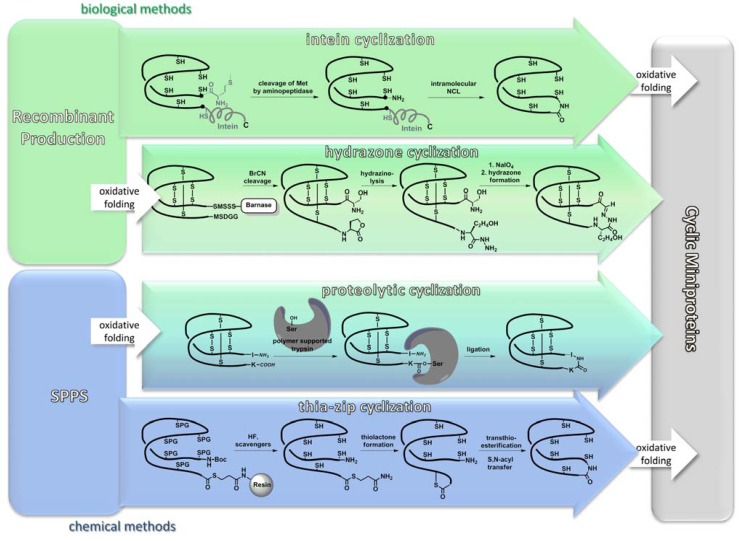
Common strategies for backbone cyclization. Biosynthetic methods are depicted in green, chemical methods in blue, hybrid strategies are shown in turquoise. References: Intein cyclization: [39,40,41]; hydrazone cyclization: [43]; proteolytic cyclization: [44,45]; thia-zip cyclization: [46,47].

In contrast to chemical synthesis, backbone cyclization (rather than oxidative folding) is the crucial step during recombinant production of cyclic cystine-knot peptides as only a few enzymes are known to catalyze the desired amide bond formation [38]. For cyclotides, intein-based cyclization methods have been recently reported [39,40,41]. Through several enzyme-catalyzed steps that include an S-N acyl shift, intein fragments are finally cleaved off and the peptide termini become condensed *via* a native amide bond (Figure 3) [39,40,41]. However, intein-mediated cyclization often lacks satisfactory yields and it remains a challenge to obtain multimilligram amounts of the respective cyclic peptide (Table 1). The recently reported production of cyclic MCoTI-I in a BL21 strain of *E. coli* indicates that high-yield synthesis is possible under fine-tuned conditions. [39,40,41,42].

**Table 1:** Selected folding mixtures for a number of cystine-knot peptides from various families.

Peptide	Type	Folding conditions	Yield/Conversion	Reference
ocMCoTI	ICK	0.5 mM HCl, 200 mM NaHCO_3_, pH = 9.1, 1–1.5 mg/L peptide.	16% ^a^	[48]
			29% ^b^	
cMCoTI	ICK	100 mM NH_4_OAc, pH = 8.5, GSH (1‑5 mM), 0.1 mg/L peptide	90% ^c^	[49]
Variants of ocMCoTI	ICK	50% MeCN in 100 mM (NH_4_)_2_CO_3_, GSH (4 eq.)	1.8–7.7% ^a^	[45]
			36–72% ^b^	
ICK toxins	ICK	GSSG/GSH (0.3 mM/0.15 mM) in 2 M urea, 100 mM Tris-HCl	3–6% ^a^	[50]
EETI-II	ICK	100 mM NH_4_OAc, pH = 9.1	>80% ^c^	[51]
Gurmarin	ICK	1. Orthogonal cysteine protecting groups 2. GSH/cystamine in 100 mM Tris-HCl, pH = 7.8	1.: 0.55% ^a^	[52]
			2.: 14.1% ^a^	
GVIA and analogues	Cono-toxin	Cysteine-selenocysteine exchange, GSSG/GSH (1 mM/2 mM)	60–78% ^c^	[53]
Cycloviolacin O2	CCK	35% DMSO, 6% Brij 35 (an oil dispersant), addition of GSH/cystamine after 24 h (2 mM/2 mM) in 100 mM Tris‑HCl, pH = 8.5	52% ^c^	[54]
Kalata B1	CCK	35% DMSO, 6%, Brij 35 (an oil dispersant), GSH/cystamine (2 mM/2 mM) in 100 mM NH_4_HCO_3_, pH = 8.5	>95% ^c^	[31]
Kalata B2	CCK	50% *i*-PrOH, GSSG/GSH (2 mM/2 mM) in 100 mM NH_4_HCO_3_, pH = 8.5	>95% ^c^	[31]
Kalata B8	CCK	50% *i*-PrOH, GSSG/GSH (2 mM/2 mM) in 100 mM NH_4_HCO_3_, pH = 8.5	>80% ^c^	[31]
Cyclic hedyotide B1	CCK	70–80% *i*-PrOH, pH = 8.5 0.033 mg/L peptide	48% ^c^	[55]
ASIP	GFCK	100 mM Tris-buffer, pH = 7.7–7.9, 1 mM EDTA, 1 M GuHCl, GSSG/GSH (1:10)	10% ^b^	[56]

^a^: yield according to resin loading; ^b^: yield according to purified linear precursor; ^c^: HPLC conversion.

As ICK peptides do not require any backbone cyclization, they can be recombinantly produced in lower organisms like bacteria or yeast [57,58,59]. It is important to mention that recombinantly produced cystine-knot peptides can be further chemically modified to yield precursors that contain a non-natural cyclization motif [43]. This issue will be detailed in the following section.

### 3.2. Chemical Synthesis

SPPS of cysteine-rich peptides has become a routine procedure and peptides comprising more than 30 amino acid residues can be obtained in good yields and enantiomeric purity. From a synthetic point of view, the most challenging issues in SPPS of cyclotides are associated with backbone cyclization (Figure 3). They will be discussed in Section 3.2.3. Nevertheless, chemical synthesis has an obvious advantage over the recombinant route as it allows one to easily incorporate non-natural elements at any desired position in the sequence. Thus, a number of non-canonic building blocks were installed in functional loops of knottins, among them a guaninyl nucleoamino acid as a conformationally restricted and less basic arginine isoster, or homoarginine and amino isobutyric acid that are known to enhance helicity of a peptide chain [48,60]. Furthermore, non-natural elements were inserted in conserved regions of knotted peptides as well. Thus, selenocysteines were installed upon SPPS replacing cysteines at crucial positions of a bracelet cyclotide which resulted in a significant improvement of folding yields [53]. Obviously, installation of non-natural functionalities not only provides an additional option for structural diversity, but also allows for the implementation of coupling sites for backbone cyclization or oligomerization [43,61].

#### 3.2.1. Chain Assembly

SPPS can be conducted by following two different general strategies. In the *tert*-butyloxycarbonyl (Boc) strategy, *α*-amino groups are protected with acid-labile Boc groups (removed *via* addition of 25% TFA), while deprotection of side chains requires stronger acidic conditions (e.g., HF, methane-sulfonic acid, *etc.*), thus ensuring “pseudo-orthogonality” of the method [62,63]. In the second orthogonal strategy the base-labile fluorenylmethyloxycarbonyl (Fmoc) moiety blocks the *α*‑amino group, whereas side chains can be deprotected with acids (e.g., TFA) [63,64]. To date, Fmoc-SPPS is often the method of choice as less corrosive and aggressive reagents are used and the elongation of the peptide chain during synthesis can be easily monitored at the Fmoc deprotection step [63]. However, the Boc strategy is still applied to SPPS of cystine-knot peptides, as it provides some obvious advantages over the Fmoc strategy [54,55,63]. Besides the incompatibility of Fmoc deprotection with the synthesis of C-terminal thioesters (Section 2.3.2), Boc chemistry often provides higher yields per coupling step [54,55,63]. Furthermore, the prices of Boc-protected amino acids in some cases are still lower in comparison to their Fmoc-protected pendants, although prices for Fmoc-protected amino acids have been decreasing continuously since the introduction of the large-scale industrial synthesis of the HIV fusion inhibitor enfuvirtide (*Fuzeon^®^*, Roche) [63,65]. The aggregation of growing peptide chains during SPPS dramatically lowers reaction yields [63]. The decreased aggregation tendency of the resin-bound peptide chain that is due to the protonated aminoterminus and backbone resulting from TFA cleavage of aminoterminal Boc groups is one major advantage of Boc- over Fmoc-SPPS [63]. Nevertheless, intermolecular aggregation, the formation of undesired secondary structures and steric hindrance can also be overcome through the usage of microwave irradiation, not only for the raise of the reaction temperature, but also for the polar peptide backbone alignment with the electromagnetic irradiation [63,66]. These effects also lead to increased reaction rates, thereby reducing formation of side-products. Moreover, prolonged reaction times in Fmoc-SPPS are outweighed through the advantage of fully automated synthesizers that can be utilized more regularly due to the usage of less aggressive reagents (although peptide synthesizers compatible with Boc-SPPS are also commercially available). In both methodologies racemization of the amino acid through deprotonating the α-hydrogen with the activator base can be easily overcome by the usage of 2,4,6-tri-methylpyridine or racemization-resistant cysteine protection as e.g., the recently reported 4-methoxy-benzyloxymethyl group [50,67]. In summary, despite Fmoc-SPPS being to date the method of choice, Boc-SPPS is a valuable back-up tool for aggregation-prone peptides or peptides with base-labile moieties which are not compatible with Fmoc-chemistry [54,55,63].

#### 3.2.2. Oxidative Folding

Oxidative folding of linear or head-to-tail cyclized precursors towards the bioactive isomer is the most important and also most critical step during synthesis of cystine-knot peptides. Significant efforts have been made to determine folding pathways and optimize oxidative folding conditions [30,31,36,49,68,69,70,71,72,73]. 

**Figure 4:**
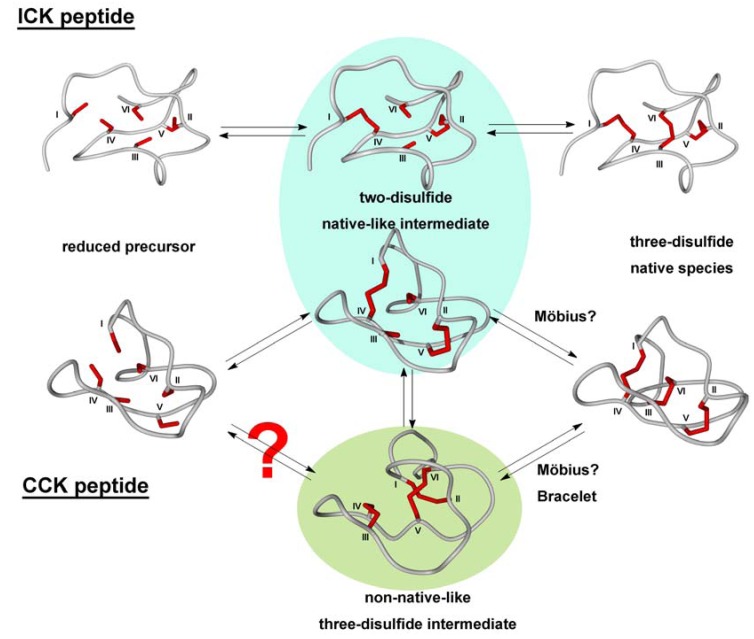
*In vitro* folding pathways of cystine-knot peptides from different families. Most ICK peptides are thought to proceed to the folded form *via* formation of two-disulfide native-like intermediate. Folding of CCK peptides may either follow a similar path or proceed *via* a non-native 3-cystine intermediate. Structures were modeled and energy-minimized with Yasara Ver. 12.4.1.

Particularly the optimization of folding conditions of cyclotides is challenging since they contain patches of hydrophobic residues on their surface, which substantially contribute to their membrane-disrupting activity [11]. These highly aggregation-prone residues tend to stick together in a non-native conformation, making therefore preorganization *via* backbone cyclization essential to obtain acceptable yields in the folding process [16,74]. CCK peptides belonging to the Möbius or bracelet family, respectively, follow different folding pathways. Möbius cystine-knot peptides rapidly form and accumulate an energetically trapped two-cystine intermediate which lacks the penetrating III,VI-disulfide, and finally fold in the native conformation either directly or *via* another, non-native three-cystine intermediate (Figure 4) [31,36]. 

The folding pathway varies with the addition of redox assistants or organic solvents [31]. However, in contrast to the members of the bracelet family, Möbius CCK peptides fold into the bioactive conformation spontaneously in good to moderate yields [31,36,38]. The *in vitro* folding of bracelet cyclotides is more challenging as their kinetic trap is not a two-cystine, but the non-native CysI-CysII, CysIII-CysIV, CysV-CysVI “ladder-like” isomer (Figure 4) [31,54]. Thus, the addition of accessory redox agents is essential. However, no recipe has been reported to date, allowing for the formation of the native form as major product [31]. To overcome these problems, cyclotides, particularly those that cannot be obtained in reasonable yields *via* random cysteine oxidation, were subjected to a stepwise folding procedure with consecutive cleavage of orthogonally protected cysteine side chains or *via* the incorporation of selenocysteines as diselenides possess a higher reduction potential [53,74,75]. Orthogonal oxidation of multiple cysteine pairs results in selective disulfide formation, thus ensuring the desired cystine pattern. From the huge repertoire of cysteine side-chain protecting groups, trityl, acetamidomethyl, S-*tert*-butyl, and *tert*-butyl are the most often used orthogonal combinations [76,77,78]. A number of cystine-knot peptides were synthesized following the strategy of regioselective disulfide formation, among them anti-malaria peptides from *Psalmopoeus cambridgei* and the ICK peptide gurmarin [52,79].

Removal of a non-critical disulfide bridge and substitution of remaining disulfides with diselenides is another way to simplify folding of multidisulfide peptides and was successfully applied to the synthesis of some conotoxines [67,80]. Interestingly, for the peptides possessing a two-disulfide pattern both cystines could be replaced on-support with isosteric cystathionine motifs without loss of bioactivity [81].

Common to twisted cyclotides, the folding pathway of ICK peptides follows a direct route resulting in a quickly formed, kinetically trapped two-disulfide intermediate [36,49,82]. This intermediate consists of two “low-energy” disulfides and therefore is vulnerable for misfolding. Thus, peptide chain preorganization and formation of correctly folded intermediates remarkably contribute to the yield and purity of the final bioactive isomer. In contrast to Möbius cyclotides, the formation of the third ring- penetrating cystine directly yields the native conformation (Figure 4) [49].

The increased hydrophilicity of the active loops in ICK peptides admits a higher degree of structural preorganization compared to the mainly hydrophobic CCK peptides, thus enhancing their folding yields. Hence, ICK peptides frequently are not macrocyclic. Nevertheless, for MCoTI variants the cyclic form displayed an improved folding behavior compared to the linear variant [27,49].

Many different folding assistants have been used in various combinations, each optimized for an individual protein or peptide. Common requirements for efficient cystine-knot peptide folding are high dilution, significant ratios of organic solvents (e.g., DMSO, *i*-PrOH) and presence of redox folding assistants (e.g., ox./red. glutathione) [30,31,36,49,68,69,70,71,72]. Despite the importance of the oxidative folding of cystine-knot peptides, particularly of those that contain grafted sequences, yields are rarely given in the present literature [54,57,58,59,83]. Moreover, missing distinctions between yield and HPLC-observed conversion rates and indications, whether they are based on resin loading or correspond to the crude or purified precursor further complicate the summarized comparison of folding yields (Table 1).

#### 3.2.3. Backbone Macrocyclization

**Figure 5:**
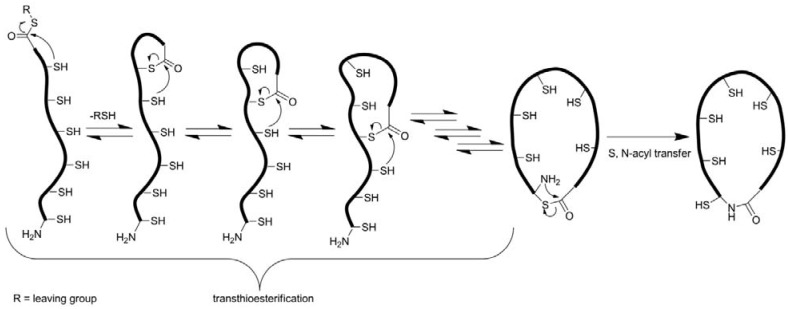
Proposed cyclization *via* native chemical ligation.

Backbone cyclization usually is accomplished *via* a so-called ‘thia-zip’ mechanism using the native chemical ligation (NCL) technology (Figure 5) [46,47,69,84,85,86,87]. To this end, a carboxyterminal leaving group, generally a thioester, must be installed. Introduction of this moiety can be achieved through thioesterification of the fully protected peptide in solution, either as a cleavable linker on the peptide resin or as a reagent during nucleophilic cleavage. For the incorporation of the thioester after chain assembly, the peptide has to be synthesized on an ‘ultra-acid-labile’ resin (e.g., a TGT resin) from which the peptide can be cleaved with all side-chain protecting groups intact. To that fully protected peptide the thiol is coupled forming the desired thioester [88]. Although this methodology is compatible with the common Fmoc-strategy of peptide synthesis, it is subjected to imponderabilities due to the unpredictable solubility of fully protected peptides, especially peptides of that size. Moreover, undesired carboxyterminal racemization may occur during synthesis [88,89]. This problem can be overcome very elegantly by choosing a glycine as aminoterminal and a cysteine as carboxyterminal residue as the site of macrocyclization, because glycine is the only non-chiral amino acid and glycine-cysteine combinations exist in a number of cystine-knot peptides (Figure 1) [88]. Installation of a thioester as on-resin cleavable linker seems more elegant, as no special modification is needed [86]. Unfortunately, piperidine that is a common reagent used in Fmoc-SPPS for N-terminal deprotection is not compatible with that linker as its nucleophilic attack at the thioester results in the cleavage of the peptide chain from the resin [86]. A combination of non-nucleophilic 1,8-diazabicyclo[5.4.0]undec-7-ene (DBU) and 1-hydroxybenzotriazole (HOBt) helps to overcome that problem on the cost of an enhanced aspartimide formation [90]. Interestingly, it has not been checked so far, whether 2-methylpiperidine could solve these problems as its utility for the synthesis of peptides with piperidine-labile tyrosine sulfate esters was demonstrated [91]. The third possibility, though not yet elaborated, might be the usage of safety-catch linkers (e.g., hydrazinobenzoyl) which can be cleaved by a respective nucleophile after suitable activation [92,93].

After successful incorporation of a carboxyterminal thioester, NCL frequently provides cyclic peptides in excellent conversions or yields, respectively (Table 2) [86,88]. Although to date the mechanism is not fully understood and not all intermediates are precisely characterized, it is commonly accepted that intramolecular thioesterifications of the internal thiol groups and the carboxyterminus take place (Figure 5) [46]. This “thia-zip” rearrangement gradually increases ring size and eventually brings both termini in close proximity. As a consequence, an irreversible *S*, *N*-acyl transfer is induced, finally leading to the cyclic product [46,84]. This model is supported by various studies, in which the aminoterminus was acetylated, a linker introduced or the ring-chain tautomeric equilibrium investigated [46,84,94]. Nevertheless, NCL-driven macrocyclization has been reported also for peptides possessing only an aminoterminal cysteine. Due to the lack of multiple thiol groups, intramolecular thialactone exchange is not possible in such molecules and ring closure takes place without zip-like rearrangements [84,85,86,95].

Despite the success of NCL as the method of choice, two alternative backbone cyclization strategies have been reported (Table 2) [43,44]. One method relies on the bacterial production of the cystine-knot peptide in *E. coli via* fusion to a carrier protein [43]. Therein, the linear precursor is fused to barnase, an RNAse from *Bacillus amyloliquefaciens*, which guides the fused protein complex into the periplasm of the Gram-negative *E. coli*, where the oxidative milieu supports folding [43,96]. Chemical head-to-tail cyclization of the folded ICK peptide was achieved through the formation of a stable *N*-C hydrazone linkage between a periodate-oxidized aminoterminal serine and a carboxyterminal hydrazide. This moiety was generated by hydrazinolysis of a homoserine lactone formed upon cyanogen bromide cleavage at a unique methionine that was present at the junction of the knottin and the carrier protein sequence (Figure 3) [43]. Recently, protease-mediated backbone cyclization was accomplished using immobilized trypsin [44,45]. Therein, a solution of chemically synthesized and correctly folded MCoTI-II was added to polymer-bound trypsin and, upon covalent binding to the active site of this protease, C- and N-termini of the cystine knot were brought into close proximity and ligated between the P1 lysine and P1’ leucine within the protease inhibiting loop (Figure 3) [44,45,97]. Conversion rates and yields for the various cyclization methods are summarized in Table 2.

**Table 2:** Selected cyclization conditions for a number of cystine-knot peptides from various families.

Peptide	Type	Cyclization reaction; conditions	Yield/Conversion	Reference
Variants of cMCoTI	ICK	Immobilized trypsin; 100 mM phosphate buffer, pH = 7.4	90–94% ^a^	[44,45]
Variants of cMCoTI	ICK	Folding and NCL as one-pot reaction; 50% GSSG (1 mM) in 100 mM carbonate buffer, 50% peptide (3 mM) in acetonitrile	63–72% ^b^	[45]
Variants of cMCoTI	ICK	Hydrazone linkage; multiple reactions from recombinantly produced barnase fusion	0.5–1 mg/L ^c^	[43]
Kalata B1	CCK	Amide bond; HBTU or BOP, respectively (1–10 eq.), DIEA in DMF	~25% ^a^	[69]
Kalata B1	CCK	NCL; 100 mM NaH_2_PO_4_, TCEP (6 eq.), pH = 7.4, 1 mg/mL peptide	100% ^a^	[69]
hB1	CCK	NCL; 100 mM NaH_2_PO_4_, 6 M GuHCl, thiophenol (100 eq.), pH = 7.5	100% ^a^	[55]
Cyclic MrIA	Cyclic cono-toxin	NCL; 100 mM Tris-HCl, pH = 7.8, 6 M GuHCl, sodium 2‑sulfonylethane sulfonate (1 mg/mL), anaerobic	100% ^a^	[98]

^a^: HPLC conversion; ^b^: yield according to purified precursor; ^c^: yield of purified cystine-knot peptide is given per liter cell culture.

#### 3.2.4. Analysis of Cystine Knots

RP-HPLC in combination with mass spectrometry, especially ESI-MS and MALDI-TOF, are commonly used for the routine analysis of cystine-knot peptides [23,31,48,50,55]. Therein, not only the polarity, but also the molecular weight are determined giving clear evidence of the quality and nature of the product [23,31,48,50,55]. For example, the progress of oxidative folding was determined through a shift in RP-HPLC retention time as well as a decreased molecular weight because of the loss of the respective number of hydrogens [23,31,48,55]. Unfortunately, this is not the final proof of correct folding as topology of the cystine connections is essential for bioactivity [51]. Therefore, determination of the correct disulfide topology is necessary which is applied routinely *via* MS-MS analytics, Edman sequencing, and protein digestion followed by subsequent MS analysis of the reaction mixture [21,24,55,95,99]. From the resulting fragments cystine connections can be deduced. Due to the small size of cystine-knot peptides, detailed structural information as the connectivity of hydrogen bonds has been collected by 2D NMR studies [25,27,82]. Nevertheless, although to date only the crystal structure of a cyclotide and an open-chain knottin have been solved, ICK peptides can be easily co-crystallized with their target enzymes [2,3,12,100].

## 4. Applications to Drug Design

Potential applications of cystine-knot peptides to drug design have been extensively reviewed [1,5,10,101]. In this section a few examples of recently developed peptides are given that highlight the advantages of their use for *in vivo* and *in vitro* targeting of disease-related molecules.

Cystine-knot peptides possess three essential characteristics which are desirable for the application as drugs: excellent stability, high affinity or inhibitory activity, and the potential for high selectivity towards the target. Additionally, these three-disulfide scaffolds provide a remarkable sequence tolerance allowing for the introduction of novel functionalities within their loop region, often without the loss of structural integrity and bioactivity [10]. Moreover, at least members of the ICK family are thought to have no cytotoxic properties and demonstrate good body clearance and tissue distribution, although these characteristics require validation for each modified candidate [1,10,101]. Interestingly, some cystine-knot peptides have been reported to be cell-penetrating [97,102].

The choice of ICK or CCK peptides as a scaffold for drug design is mainly guided by the natural target molecule, although several instances of engineering towards completely different targets have been reported [59,103,104]. As natural ICK peptides usually target trypsin-like proteases, they are optimal starting points for obtaining potent inhibitors against other disease-related serine proteases [9,23,26]. Engineered “imino-cyclotides” combining the ICK backbone with a non-natural hydrazone cyclization motif have been reported to inhibit human mast cell tryptase *β*, a protease of interest as a therapeutic target for the treatment of inflammatory disorders and allergic asthma [43]. 

For tumor targeting, several transmembrane serine proteases which are known to be overexpressed in cancer cells are also valuable targets [105,106,107,108]. 

Most interestingly, Agouti-related miniprotein (AgRP), an acyclic four-cystine knottin, has recently been modified with an RGD peptide motif towards binding of cancer-dependent integrins and the resulting constructs were used for radio imaging *in vivo* [59,103,109]. A prototype for the engineering of miniproteins towards variants with antiviral activity is the HIV entry inhibitor CD4M47^[Phe]^. Here, the miniprotein Leiurotoxin I from the deathstalker scorpion *Leiurus quinquestriatus hebraeus* was used as a structural scaffold [110,111,112,113]. Several rounds of directed evolution and rational design resulted in an optimized binding towards gp120 of the viral particle of HIV, thus inhibiting cell entry [110,111,112]. CCK peptides in most cases have shown antiviral or bactericidal activity in their wild-type form, but to date only some of them have been grafted towards new bioactivities [6,20,114,115].

## 5. Conclusions and Outlook

Cystine-knot peptides are defined through their unique architecture which endows them with an extremely high stability and sequence tolerance resulting in promising scaffolds for drug development and chemical genetics. Current synthetic problems, oxidative folding and backbone cyclization, depend on whether cystine-knot peptides are recombinantly produced or chemically synthesized. Head-to-tail macrocyclization is problematic for recombinantly produced peptides, and formation of the three-disulfide pattern for those chemically synthesized. As for large-scale industrial processes, *in vivo* synthesis may become a cost-effective alternative to chemical synthesis, but microorganisms and their respective production conditions need further optimization. Novel pharmaceuticals based on cystine-knot peptides may find their way to clinical trials in the next couple of years. Continuously reported improvements in their functionalization towards modulators of disease-relevant targets in combination with the increasing number of publications for both chemical synthesis and recombinant production provide excellent future prospects.
